# RNA-seq analysis of gene expression changes during pupariation in *Bactrocera dorsalis* (Hendel) (Diptera: Tephritidae)

**DOI:** 10.1186/s12864-018-5077-z

**Published:** 2018-09-21

**Authors:** Er-Hu Chen, Qiu-Li Hou, Wei Dou, Dan-Dan Wei, Yong Yue, Rui-Lin Yang, Shuai-Feng Yu, Kristof De Schutter, Guy Smagghe, Jin-Jun Wang

**Affiliations:** 1 Key Laboratory of Entomology and Pest Control Engineering, College of Plant Protection, Southwest University, Chongqing, 400715 China; 2 Academy of Agricultural Sciences, Southwest University, Chongqing, 400715 China; 3 Department of Plants and Crops, Ghent University, 9000 Ghent, Belgium

**Keywords:** *Bactrocera dorsalis*, Pupariation, Metamorphosis, RNA-Seq, Gene expression

## Abstract

**Background:**

The oriental fruit fly, *Bactrocera dorsalis* (Hendel) has been considered to be one of the most important agricultural pest around the world. As a holometabolous insect, larvae must go through a metamorphosis process with dramatic morphological and structural changes to complete their development. To better understand the molecular mechanisms of these changes, RNA-seq of *B. dorsalis* from wandering stage (WS), late wandering stage (LWS) and white puparium stage (WPS) were performed.

**Results:**

In total, 11,721 transcripts were obtained, out of which 1914 genes (578 up-regulated and 1336 down-regulated) and 2047 genes (655 up-regulated and 1392 down-regulated) were found to be differentially expressed between WS and LWS, as well as between WS and WPS, respectively. Of these DEGs, 1862 and 1996 genes were successfully annotated in various databases. The analysis of RNA-seq data together with qRT-PCR validation indicated that during this transition, the genes in the oxidative phosphorylation pathway, and genes encoding P450s, serine protease inhibitor, and cuticular proteins were down-regulated, while the serine protease genes were up-regulated. Moreover, we found some 20-hydroxyecdysone (20E) biosynthesis and signaling pathway genes had a higher expression in the WS, while the genes responsible for juvenile hormone (JH) synthesis, degradation, signaling and transporter pathways were down-regulated, suggesting these genes might be involved in the process of larval pupariation in *B. dorsalis*. For the chitinolytic enzymes, the genes encoding chitinases (chitinase 2, chitinase 5, chitinase 8, and chitinase 10) and chitin deacetylase might play the crucial role in the degradation of insect chitin with their expressions significantly increased during the transition. Here, we also found that chitin synthase 1A might be involved in the chitin synthesis of cuticles during the metamorphosis in *B. dorsalis*.

**Conclusions:**

Significant changes at transcriptional level were identified during the larval pupariation of *B. dorsalis*. Importantly, we also obtained a vast quantity of RNA-seq data and identified metamorphosis associated genes, which would all help us to better understand the molecular mechanism of metamorphosis process in *B. dorsalis*.

**Electronic supplementary material:**

The online version of this article (10.1186/s12864-018-5077-z) contains supplementary material, which is available to authorized users.

## Background

The oriental fruit fly, *Bactrocera dorsalis* (Hendel), is a highly invasive species which has been found in India, East Asia and the Pacific region. This insect can cause significant economic losses to many commercially important tropical and subtropical crops, especially fruits, including citrus, banana, carambola, and mango [1, 2]. Because of being highly polyphagous and invasive, *B. dorsalis* has been considered as one of the most notorious pests of agricultural fruit around the world. To prevent its expansion to new host plants and geographic areas, many countries have imposed strict quarantine restrictions against *B. dorsalis* [3].

As a dipteran insect, *B. dorsalis* is a typical holometabolous insect which needs to go through larval-pupal metamorphosis to molt into adults. The metamorphosis of flies is a complex process accompanied with drastic morphological and physiological changes. Larval pupariation is the critical process during the larval-pupal development, in which the soft cuticle of a wandering larva is transformed into a hard puparium, this process involves immobilization, cuticular shrinkage, anterior retraction, longitudinal body contraction, and tanning of the cuticle [4, 5]. It has also been reported that most of the larval tissues (integument, midgut, and fat body) must undergo a series of developmental events involving programmed cell death, and cell proliferation and differentiation to remodel structures of insects [6].

Juvenile hormone (JH) is an important hormone in insects, which coordinates with 20-hydroxyecdysone (20E) to regulate growth and development [6]. While both of these hormones are present at larval stages, they have opposing roles in the development. For example, JH maintains insects at larval stage, in contrast, 20E induces metamorphosis at the end of the last larval stage when JH titers decrease [7, 8]. The regulation of metamorphosis by hormones has been widely studied in many insects including *Drosophila melanogaster* [9], *Bombyx mori* [10], and *Tribolium castaneum* [11]. These results indeed show that the regulation of the JH titer in the hemolymph plays an important role in precisely mediating the process of larval-pupal metamorphosis in insects [12].

In holometabolous insects, the larval cuticle is degraded and a new cuticle of the pupal counterpart is secreted by the underlying epidermal cells during the larval-pupal metamorphosis. As chitin is the crucial component of the cuticle, the insect metamorphosis is largely dependent on chitin degradation and synthesis, which is strictly coordinated and occurs almost simultaneously within the process of molting and metamorphosis [13]. The chitin biosynthesis pathway is catalyzed by a series of enzymes, which starts with trehalose, then hexokinase, glucose-6-phosphate isomerase (G-6-P-I), glutamine-fructose-6-phosphate aminotransferase (G-F-6-P-A), glucosamine-6-phosphate N-acetyltransferase (G-6-P-A), phosphoacetylglucosamine mutase (PM), UDP-N-acetylglucosamine pyrophosphorylase (UDP-N-A-P), and chitin synthase [14]. For the chitin catabolic pathway, chitinase, β-N-acetylglucosaminidase, and chitin deacetylase are the three main enzymes that are involved in chitin degradation, which hydrolyze chitin into oligosaccharides, and further degrade these oligomers to monomers [15].

In previous studies, the gene expression patterns during metamorphosis process have been reported in many insects, including *B. mori*, *Manduca sexta*, and *Spodoptera litura* [16–18], however, little is known about the molecular mechanism of metamorphosis in *B. dorsalis*. In this paper, we elucidate the larval pupariation process by analyzing the transcriptome of *B. dorsalis* using RNA-seq at three developmental stages including the wandering stage (WS), late wandering stage (LWS, the body contraction just before pupariation), and white puparium stage (WPS). The use of RNA-seq is already well established for the analysis of gene expression in *B. dorsalis* [3, 19, 20], and the availability of a well annotated genome of *B. dorsalis* (NCBI Assembly: ASM78921v2), allowed us to identify the genes differentially expressed during the different developmental stages. The results obtained through the RNA-seq analysis were further confirmed by real-time quantitative PCR. For an in-depth analysis, we focused on the genes related to oxidative phosphorylation, drug metabolism-cytochrome P450, amino acids metabolism, 20E, JH, and chitin synthesis and degradation.

## Methods

### Insect and RNA isolation

**Fig. 1:**
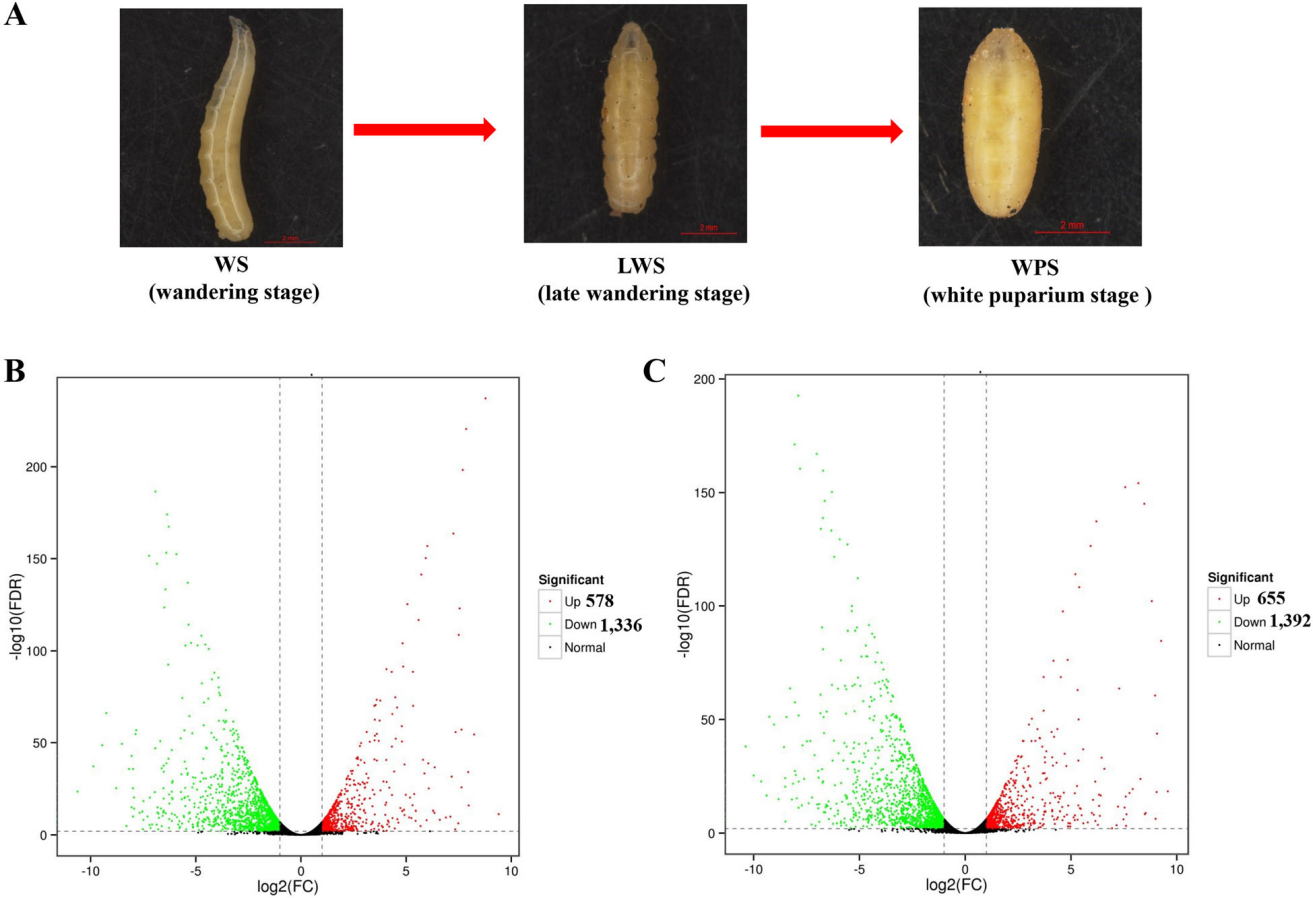
**a** The morphology of three developmental stages including wandering stage (WS), late wandering stage (LWS), and white puparium stage (WPS) during the pupariation of *B. dorsalis*. Numbers of differentially expressed genes (DEGs, FDR < 0.01 and |log2 ratio| ≥ 1), the up-regulated genes were represented by a red dot and down-regulated genes by a green dot. **b** DEGs of WS vs. LWS (**c**) and WS vs. WPS

The stock colony of *B. dorsalis* was collected and reared according to the method described in our previously study [21]. Briefly, *B. dorsalis* was reared at 27 ± 1 °C and 70 ± 5% relative humidity with a photoperiod regime of 14:10 h light/darkness. The larvae were reared on an artificial diet containing corn, yeast powder, wheat flour as well as sucrose. A plastic basin was provided for pupation of the 3rd instar larvae. Three developmental stages, including the wandering stages (WS), late wandering stage (LWS), and white puparium stage (WPS), were collected (Fig. 1a). The 3rd-instar larvae that just jump into sand were referred as WS, the LWS was identified as the body contraction just before pupariation, and the insects that had just completed the formation of white puparium were defined as the WPS. Three replicates were performed for each stage (WS, LWS, and WPS) and each replicate contained eight flies. Total RNA was extracted by the RNeasy plus Micro Kit (Qiagen GmbH, Hilden, Germany) following the manufacturer’s instructions. In brief, the absorbance at 260 nm was used to quantify the RNA by a NanoVue UVVis spectrophotometer (GE Healthcare Bio-Science, Uppsala, Sweden). The absorbance ratio of OD260/280 and OD260/230 were used to test the purity of all RNA samples. The integrity of RNA was assessed by 1% agarose gel electrophoresis.

### cDNA library construction and RNA-seq sequencing

A total amount of 1 μg RNA per sample was used as input material for the RNA sample preparations. Sequencing libraries were generated using NEBNext UltraTM RNA Library Prep Kit for Illumina (NEB, Ipswich, MA, USA) following manufacturer’s recommendations and index codes were added to attribute sequences to each sample.

Briefly, the poly-T oligo-attached magnetic beads were used for the purification of total RNA. Fragmentation was applied by using divalent cations under elevated temperature in NEBNext first strand synthesis reaction buffer (5X). The random hexamer primer and M-MuLV reverse transcriptase were used to synthesize the first strand cDNA. The second strand cDNA synthesis was subsequently performed using DNA Polymerase I and RNase H. Then, the remaining overhangs were translated into blunt ends by using exonuclease/polymerase activities. The NEBNext adaptor with hairpin loop structure was ligated for further hybridization after adenylation of the 3′ ends of the DNA fragments. The purification of the library fragments was performed using the AMPure XP system (Beckman Coulter, Beverly, MA, USA) to select cDNA fragments of preferentially 240 bp in length. Afterwards, 3 μl USER Enzyme (NEB) was used with size-selected, adaptor-ligated cDNA at 37 °C for 15 min followed by 5 min at 95 °C before PCR. The PCR was conducted with fusion high-fidelity DNA polymerase, universal PCR primers and index (X) Primer. Finally, PCR products were purified (AMPure XP system) and the Agilent Bioanalyzer 2100 system (Santa Clara, CA, USA) was used to evaluate library quality.

The clustering of the index-coded samples was performed on a cBot cluster generation system using the TruSeq PE Cluster Kit v4-cBot-HS (Illumina) according to the manufacturer’s instructions. After cluster generation, the library preparations were sequenced on an Illumina HiSeq Xten platform and paired-end reads were generated.

### Analysis of RNA-seq data

Raw data (raw reads) of fastq format were firstly processed through in-house PERL scripts. In this step, clean data (clean reads) were obtained by removing reads containing adapters, reads containing poly-N and low quality reads from raw data. At the same time, Q20, Q30, GC-content, and sequence duplication level of the clean data were calculated. These clean reads were then mapped to the reference genome sequence. Only reads with a perfect match or one mismatch were further analyzed and annotated based on the reference genome. Tophat2 tools soft were used to map with the reference genome [22]. The gene expression levels were further quantified by FPKM (Fragment Per Kilobase of exon model per million mapped reads) method [23]. To confirm the pairs of RNA-seq results are faithful replicates, the FPKM between the biological replications was analyzed by Pearson correlation [24]. The formula is shown as follow:

$$ \mathrm{FPKM}=\frac{\mathrm{cDNA}\ \mathrm{Fragments}}{\mathrm{Mapped}\ \mathrm{Fragments}\ \left(\mathrm{Millions}\right)\times \mathrm{Transcript}\ \mathrm{Length}\ \left(\mathrm{kb}\right)\ } $$

In this study, differential expression analysis of two developmental stages was performed using the DESeq method. DESeq provides statistical routines for determining differential expression in digital gene expression data using a model based on the negative binomial distribution [25]. The resulting *P* values were adjusted using the Benjamini and Hochberg’s approach for controlling the false discovery rate (FDR). Those genes that had a two fold difference (the absolute value of log2 ratio ≥ 1) between two stages and an FDR less than 0.01 were identified as differentially expressed genes (DEGs). Gene function was annotated based on the following databases: NCBI non-redundant protein sequences (Nr), Protein family (Pfam), Clusters of Orthologous Groups of proteins (COG), a manually annotated and reviewed protein sequence database (Swiss-Prot), evolutionary genealogy of genes: Non-supervised Orthologous Groups (eggNOG), Kyoto Encyclopedia of Genes and Genomes (KEGG Ortholog database), and Gene Ontology (GO).

GO analysis of DEGs was constructed by the Blast2GO method [26]. Namely, the DEGs were mapped into GO terms in the database (www.geneontology.org) and calculated the gene numbers for each term. Then, significantly enriched terms were identified based on ‘GO::TermFinder’ (https://www.yeastgenome.org/goTermFinder). For the COG annotation, the DEGs between two developmental stages (WS vs. LWS, and WS vs. WPS) were translated into amino acid sequences using the virtual ribosome package. We also used the batch web CD search tool (http:// https://www.ncbi.nlm.nih.gov/Structure/bwrpsb/bwrpsb.cgi) to assign COG groups. KEGG is a database resource for understanding high-level functions and utilities of the biological system, such as the cell, the organism and the ecosystem, and for molecular-level information, especially large-scale molecular datasets generated by genome sequencing and other high-throughput experimental technologies (http://www.genome.jp/kegg/) [27]. We used KOBAS software to test the statistical enrichment of differential expression genes in KEGG pathways [28]. Significant enrichments of the transcripts were tested by calculating the *p*-value from a Bonferroni correction at the background level of all GO, COG and KEGG classifications and we take the corrected *p*-value < 0.05 as a threshold.

### Quantitative real-time polymerase chain reaction (qRT-PCR)

In this study, qRT-PCR was carried out to validate the expression of the target genes. Total RNA was extracted from the three developmental stages of WS, LWS, and WPS using TRIzol reagent. As described above, the total RNA from each stage was used for the first strand cDNA synthesis by using reverse transcriptase (Takara) and oligo d (T) primer (Takara, Dalian, China). Then, Novostar-SYBR Supermix (Novoprotein, Shanghai, China) was used for qRT-PCR with a 10 μl reaction system. This system consisted of 0.5 μl of cDNA samples (approximately 200 ng/mL), 0.3 μl of each primer (10 mM), 5 μl of Supermix, and 3.9 μl of nuclease-free water. The qRT-PCR was performed with an initial 2 min denaturation at 95 °C, then 40 cycles at 95 °C for 15 s and at 60 °C for 30 s in a Mx3000P thermal cycler (Stratagene, La Jolla, CA, USA). To ensure product specificity, the melting curve was analyzed for all reactions from 60 °C to 95 °C at the end of PCR reactions. The primers used in this study were listed in Additional file 1: (Table S1). The *α-tubulin* (GenBank accession number: GU269902) has the excellent stability for temporal and spatial distribution of *B. dorsalis*, and was therefore used as an internal reference gene [29, 30]. Based on normalization with the reference gene, the 2^−ΔΔCT^ method was used to determine the expression changes during the larval pupariation [31]. All experiments were performed in three biological replicates, the data was analyzed by one-way analysis of variance (ANOVA), and the means were separated by a least significant difference (LSD) test (*P* < 0.05) via SPSS 16.0 software (SPSS Inc., Chicago, IL, USA).

## Results

### Overview of the RNA-seq data

To investigate the expression changes of genes during the larval pupariation process, we performed RNA-Seq analysis on three developmental stages (WS, LWS, and WPS) of *B. dorsalis* with its genome as a reference. The samples from three stages were sequenced in triplicate and a total of 74.62 Gb clean data was obtained. After the strict quality control, more than 99.35% of the clean data was retained. Between 70.09 and 74.44% of the reads could be mapped onto the *B. dorsalis* genome. The sequencing data has an average GC content of 41.54% and a Q30 of 91.61% (Additional file 2: Table S2).

### Analysis of differentially expressed genes (DEGs)

FPKM was calculated and standardized for the analysis of gene expression, and similar patterns of FPKM density were obtained for each sample of *B. dorsalis*, suggesting high reproducibility of the transcriptome analysis of each developmental stage (Additional file 3: Figure. S1). In total 11,721 genes were identified from the 9 samples and their gene expression profiles are shown in Additional file 4: (Table S3). There were 11,301 genes that could be annotated in *B. dorsalis* genome, this is 83.76% of the total predicted genes. We also found 420 genes that could not be annotated in *B. dorsalis* genome, and were viewed as the novel genes.

In this study, we found significant gene expression changes (two fold difference between two stages with an FDR less than 0.01) accompanying pupariation in *B. dorsalis* (Fig. 1b and c). From WS to LWS (WS vs. LWS), 1914 genes were identified as differentially expressed, among which 578 were up-regulated and 1336 were down-regulated (Fig. 1b). From WS to WPS (WS vs. WPS), 2047 genes were identified as differentially expressed, of which 655 were up-regulated and 1392 were down-regulated (Fig. 1c). However, from LWS to WPS (LWS vs. WPS), only 160 genes were detected differentially expressed with 65 were up-regulated and 95 were down-regulated (Additional file 5: Figure. S2), suggesting less changes at molecular levels between these two stages. Therefore, we focused on the analysis of DEGs between WS vs. LWS and WS vs. WPS.

### GO, COG and KEGG analysis of differentially expressed genes (DEGs)

To explore the potential function of the DEGs during pupariation, seven databases were used, including COG, GO, KEGG, Nr, Pfam, Swiss-Prot, and eggNOG (Additional file 6: Figure. S3). As there was the large overlap of DEGs between WS vs. LWS and WS vs. WPS, similar annotations were identified for each database, and totally 1862 (Additional file 7: Table S4) and 1996 (Additional file 8: Table S5) genes were successfully annotated, respectively. For the GO classification, the DEGs of two comparisons (WS vs. LWS and WS vs. WPS) were both divided into three ontologies: biological process, cellular component, and molecular function, including 49 annotations for each comparison (Additional file 9: Figure. S4 and Additional file 10: Figure. S5). Furthermore, a COG functional classification was performed to compare the enriched functional categories of the DEGs between WS and LWS or WS and WPS using the rpstBlastn program [32]. When mapping all the DEGs into the COG database, a total of 624 (WS vs. LWS) and 667 (WS vs. WPS) genes were annotated, which had a similar COG classification (Additional file 11: Figure. S6). The two DEGs profiles were each annotated into 22 COG categories, and the largest group in the cluster was ‘general function prediction only’, followed by ‘Amino acid transport and metabolism’, ‘Carbohydrate transport and metabolism’, ‘Inorganic ion transport and metabolism’, ‘Secondary metabolites biosynthesis, transport and catabolism’, ‘Lipid transport and metabolism’, ‘Signal transduction mechanisms’, and ‘Energy production and conversion’.

**Fig. 2:**
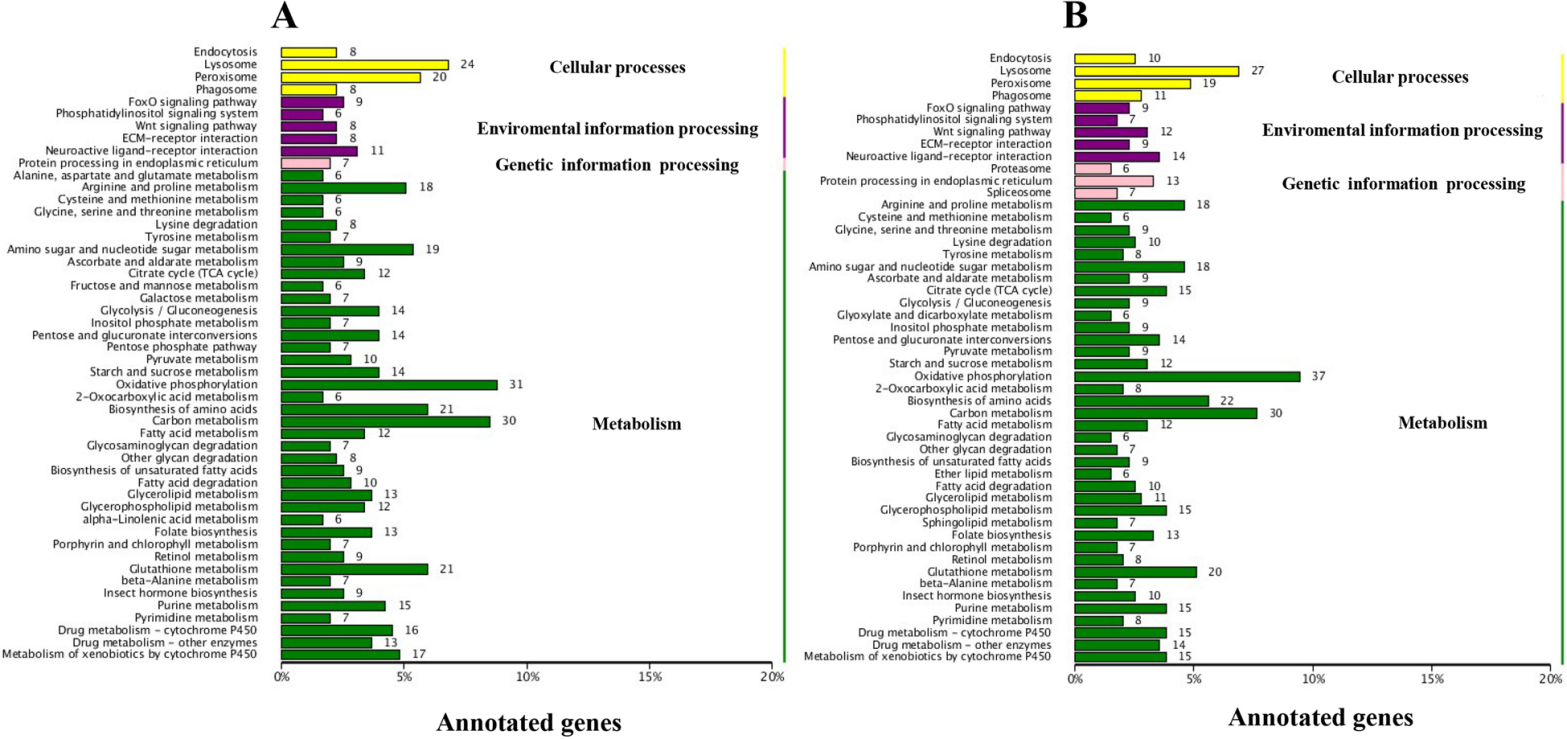
KEGG pathway classification for differentially expressed genes (DEGs) during the pupariation of *B. dorsalis*. **a** KEGG pathway classification for DEGs between wandering stage (WS) and late wandering satge. **b** KEGG classification of DEGs in WS and white puparium stage (WPS)

**Fig. 3:**
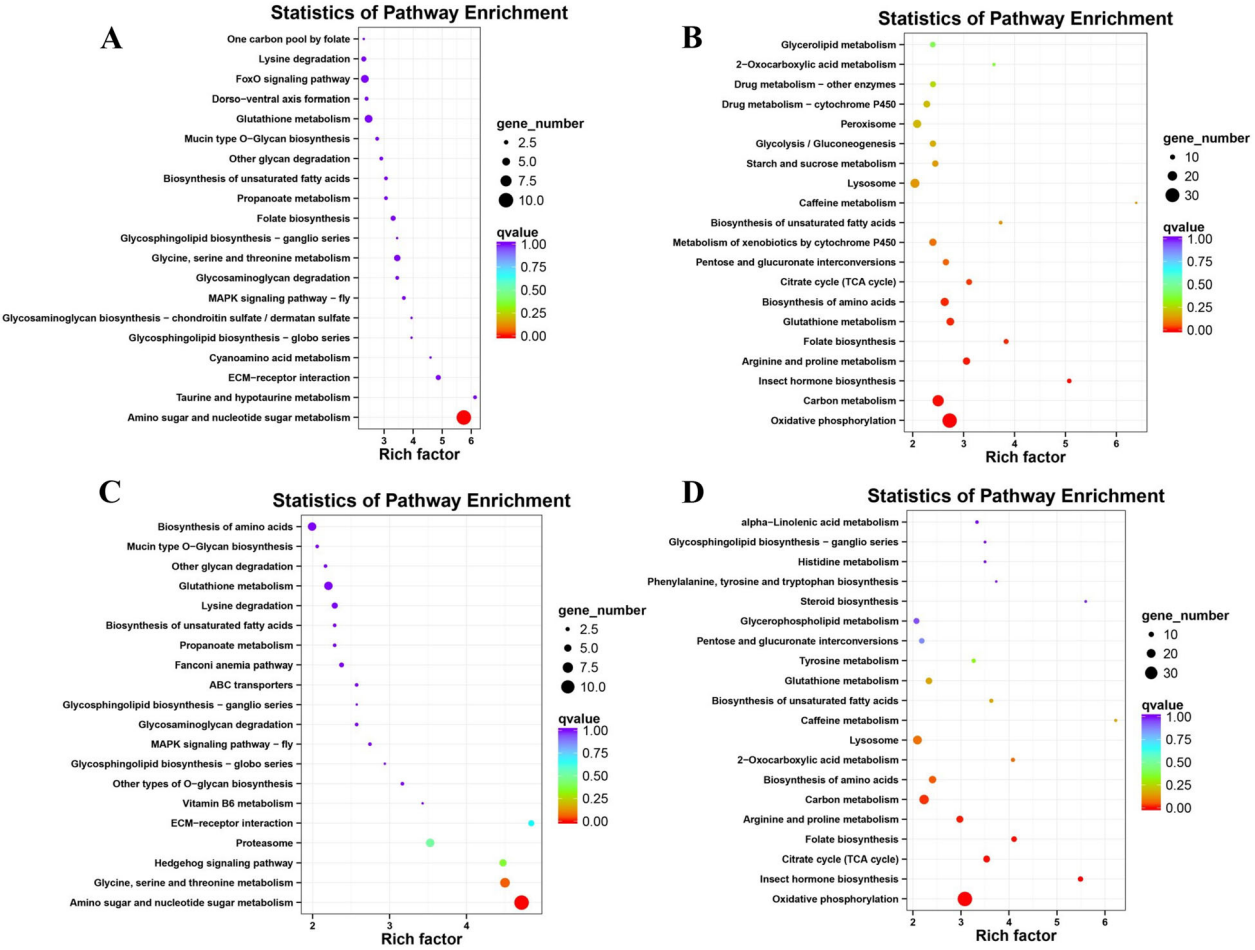
KEGG significant enrichment analysis for differentially expressed genes (DEGs). **a** KEGG significant enrichment analysis for the up-regulated genes between wandering stage (WS) and late wandering stage (LWS). **b** KEGG significant enrichment analysis for the down-regulated genes that between WS and LWS. **c** KEGG significant enrichment analysis for the up-regulated genes between WS and white puparium stage (WPS). **d** KEGG significant enrichment analysis for the down-regulated genes between WS and WPS

Here, the DEGs of WS vs. LWS and WS vs. WPS were each mapped to the KEGG pathways, respectively, a total of 658 and 722 genes were classified into 50 pathways (Fig. 2). Similar to the COG annotation, the metabolism pathways is also the largest classification in the KEGG analysis, which contained ‘oxidative phosphorylation’, ‘carbon metabolism’, ‘biosynthesis of amino acids’, ‘glutathione metabolism’, and ‘amino sugar and nucleotide sugar metabolism’. Furthermore, two pathways of ‘drug metabolism-cytochrome P450’ and ‘metabolism of xenobiotics by cytochrome P450’ were involved in metabolism classification as well (Fig. 2). For the cellular processes and environmental information processing, the ‘endocytosis’, ‘lysosome’, ‘peroxisome’ and ‘phagosome’, and the ‘FoxO signaling’, ‘phosphatidylinositol signaling system’, ‘wnt signaling’, ‘ECM-receptor interaction’ and ‘neuroactive ligand-receptor interaction’ pathways were identified respectively. As for the genetic information processing, only the ‘protein processing in endoplasmic reticulum’ pathway was annotated for DEGs (WS vs. LWS), and three pathways including ‘proteasome’, ‘protein processing in endoplasmic reticulum’ and ‘spliceosome’ were annotated for DEGs (WS vs. WPS). Importantly, the KEGG pathway enrichment analysis showed that the ‘amino sugar and nucleotide sugar metabolism’ pathway was significantly enriched for the up-regulated genes (WS vs. LWS and WS vs. WPS) (Fig. 3a and c), and the ‘oxidative phosphorylation’ and ‘insect hormone biosynthesis’ pathways were significant enriched for the down-regulated genes (Fig. 3b and d). Generally speaking, the GO, COG and KEGG analysis give us an overview of function of DEGs, and the pathways described above might play an important role in the process of pupariation in *B. dorsalis*.

### Changes of the gene expressions in the oxidative phosphorylation pathway

**Fig. 4:**
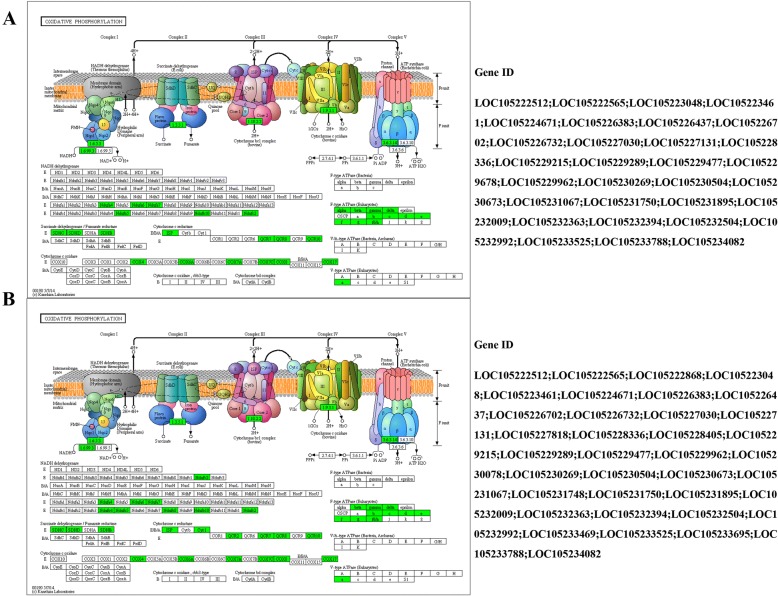
The KEGG pathway of the oxidative phosphorylation pathway responds to pupariation, and genes highlighted in green are enriched and down-regulated. **a** wandering stage (WS) vs. late wandering stage. **b** WS vs. white puparium stage

The genes in the oxidative phosphorylation pathway were significantly down-regulated during the pupariation of *B. dorsalis*, specifically the genes encoding NADH dehydrogenase, succinate dehydrogenase, cytochrome c reductase, cytochrome c oxidase, and ATPase (Fig. 4).

### Changes in expression level of P450 genes

A large number of P450 genes were significantly down-regulated during the larval pupariation in *B. dorsalis*, especially for CYP4 and CYP6 members (Table 1). Interestingly, we also found three CYP3 genes that were significantly up-regulated (Table 1).

**Table 1:** Expression level of the P450 genes during the pupariation in *Bactrocera dorsalis*

Gene ID	WS-FPKM	LWS-FPKM	WPS-FPKM	WS vs LWS	WS vs WPS	Blast nr
Down-regulated genes						
LOC105230198	6.14	0.11	0.25	−6.10	−4.67	cytochrome P450 6a13
LOC105233810	1577.65	48.39	76.59	−5.02	−4.37	cytochrome P450 6 g2
LOC109579301	92.94	4.84	3.95	−4.54	−4.27	cytochrome P450 6 g1-like
LOC105222599	405.68	20.38	16.49	−4.39	−4.68	cytochrome P450 6 g1-like
LOC105228198	3.01	0.20	0.11	−4.14	−4.81	cytochrome P450 6a9-like
LOC105233910	59.36	3.82	18.24	−3.95	− 1.70	cytochrome P450 4c3
LOC105230949	152.70	10.16	7.75	−3.91	−4.28	cytochrome P450 6a14
LOC105225363	190.01	13.39	15.48	−3.85	−3.64	cytochrome P450 9b2-like
LOC105230203	61.83	5.34	3.85	−3.53	−4.00	cytochrome P450 6a9-like
LOC105230201	22.22	2.09	0.98	−3.46	−4.58	cytochrome P450 6a21
LOC105231082	81.51	8.77	5.23	−3.24	−4.01	cytochrome P450 12e1, mitochondrial
LOC105232227	8.40	0.92	1.61	−3.23	−2.36	cytochrome P450 311a1
LOC105231049	1370.38	159.16	56.06	−3.17	−4.67	cytochrome P450 304a1
LOC105228053	16.88	1.96	2.48	−3.15	−2.78	cytochrome P450 4ad1
LOC105231113	205.39	23.07	9.89	−3.08	−4.06	cytochrome P450 12e1
LOC105224924	39.70	5.56	11.36	−2.87	−1.82	cytochrome P450 18a1
LOC105231454	27.69	5.56	10.06	−2.36	− 1.44	cytochrome P450 4d8
LOC105230935	48.89	10.80	13.69	−2.19	−1.85	cytochrome P450 6d5
LOC105230199	12.80	2.85	3.38	−2.16	−1.91	cytochrome P450 317a1
LOC105230713	7.86	1.78	1.91	−2.15	−2.03	cytochrome P450 301a1, mitochondrial
LOC105226210	53.21	13.39	10.29	−2.02	−2.39	cytochrome P450 12c1, mitochondrial
LOC109580018	17.03	4.82	2.61	−2.01	−3.05	cytochrome P450 313a4
LOC105234039	254.72	70.14	60.75	−1.88	−2.16	cytochrome P450 309a1
LOC105227474	221.04	60.58	48.65	−1.84	−2.15	cytochrome P450 6a2-like
LOC105228051	79.19	23.67	23.14	−1.76	−1.80	cytochrome P450 4e2-like
LOC105225824	50.18	10.47	12.11	−1.73	−1.66	cytochrome P450 9f2
LOC105226035	170.22	52.36	53.18	−1.71	−1.70	cytochrome P450 6d4
LOC105225364	262.08	82.38	56.90	−1.68	−2.24	cytochrome P450 9b2-like
LOC105225361	30.28	9.72	15.56	−1.68	−1.01	cytochrome P450 9 h1
LOC105230202	30.33	9.71	10.32	−1.66	−1.54	cytochrome P450 6a21
LOC105229174	37.97	12.68	8.74	−1.64	−2.18	cytochrome P450 4p1-like
LOC105227668	27.04	8.80	6.78	−1.62	−2.00	cytochrome P450 4aa1
LOC105224875	10.38	4.78	4.89	−1.11	−1.05	cytochrome P450 302a1, mitochondrial
Up-regulated genes						
LOC105226220	1.37	13.26	61.18	3.28	5.50	cytochrome P450 12b2, mitochondrial
LOC105227996	5.00	29.60	30.28	2.81	2.77	cytochrome P450 313a4
LOC105227639	77.80	233.28	440.26	1.57	2.50	cytochrome P450 12a4, mitochondrial
LOC105226219	45.76	134.86	207.65	1.56	2.18	cytochrome P450 12b1, mitochondrial-like
LOC105230977	6.85	13.84	13.31	1.22	1.14	cytochrome P450 313a4
LOC105231048	541.23	1207.42	1104.91	1.14	1.03	cytochrome P450 304a1

The criteria applied for significance difference are FDR ≤ 0.01, and estimated absolute |log2Ratio| ≥ 1

### Changes of the gene expressions in the 20-hydroxyecdysone (20E) and juvenile hormone (JH) pathways

**Fig. 5:**
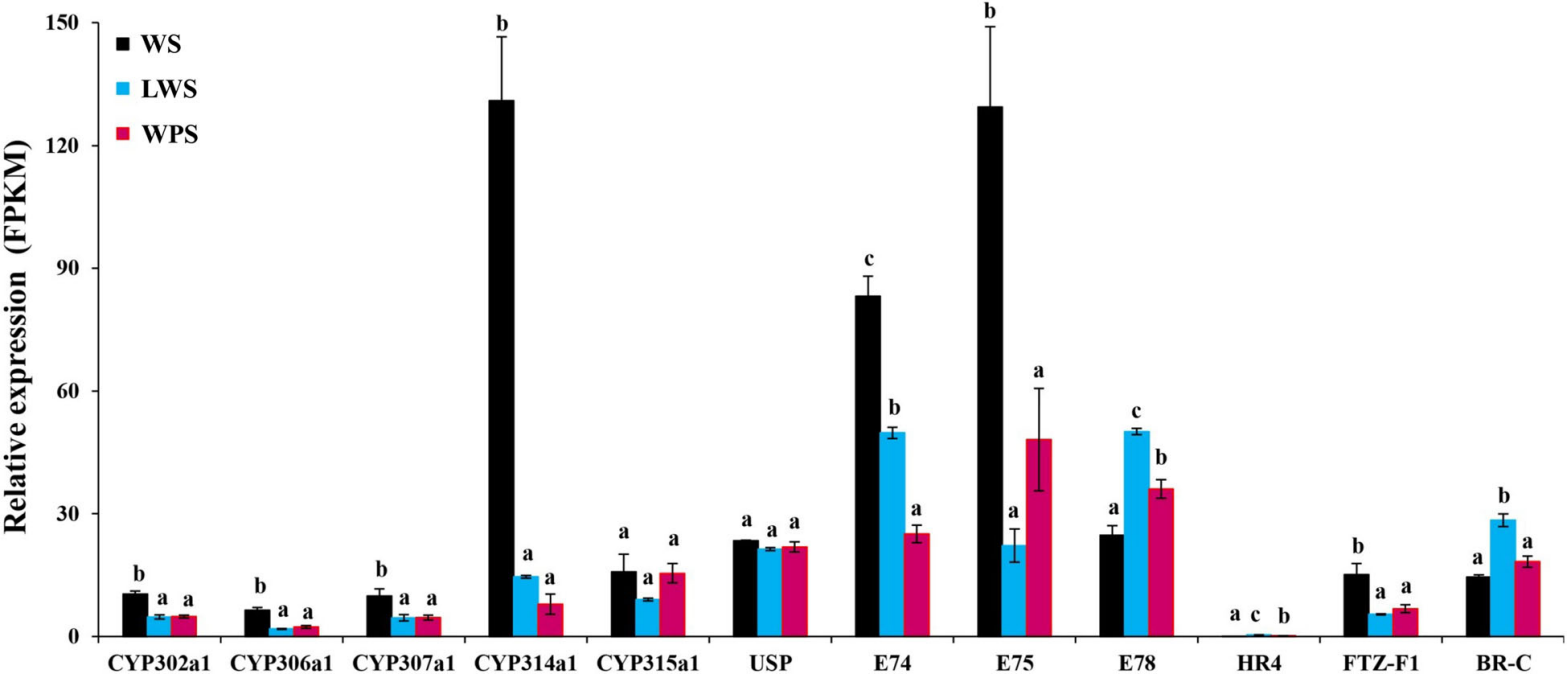
The expression patterns (FPKM value) of five 20E biosynthesis and seven signaling pathway genes during the pupariation in *Bactrocera dorsalis*. Three replications were conducted, and the data are presented as mean ± SE. Significant differences among the three treatments were analyzed by one-way analysis. Bars with different letters above them differ significantly at *P* < 0.05

Four genes encoding CYP302a1, CYP306a1, CYP307a1 and CYP314a1, involved in 20E biosynthesis, and three 20E signaling pathway genes encoding E74, E75, and FTZ-F1 had the highest expressions in the wandering stage (Fig. 5 and Additional file 12: Table S6). The JH synthesis pathway contains a series of enzymatic reactions and in total 10 genes could be detected during the larval metamorphosis in *B. dorsalis* (Table 2). Especially the expression of the three genes encoding farnesol dehydrogenase (FD), farnesoic acid O-methyl transferase (FAOMT) and juvenile hormone acid O-methyltransferase (JHAOM) was significantly decreased in the process of pupariation. Interestingly, we found three key enzymes (JH epoxide hydrolase, JH diol kinase, and JH esterase) involved in the degradation of JH that were significantly down-regulated in *B. dorsalis* (Table 2). As the key genes in the JH signal transduction pathway, the gene expressions of methoprene-tolerant (Met), Krüppel homolog 1 (Kr-h1) and steroid receptor coactivator (SRC) were significantly down-regulated during the pupariation in *B. dorsalis* as well (Table 2). Moreover, the JH transport pathway was significantly blocked, with the transcript levels of the JHBP genes significantly decreased during the larval metamorphosis in *B. dorsalis* (Table 2).

**Table 2:** Expression level of the juvenile hormone related genes during the pupariation in *Bactrocera dorsalis*

Gene ID	WS-FPKM	LWS-FPKM	WPS-FPKM	WS vs LWS	WS vs WPS	Blast nr
juvenile hormone synthesis pathway						
LOC105226875	158.49	193.63	216.66	+	+	Acetoacetyl-CoA thiolase
LOC105227740	11.83	6.01	5.89	–	− 1.03*	3-Hydroxy-3-methylglutaryl-CoA synthase
LOC105226753	2.91	4.38	4.36	+	+	3-Hydroxy-3-methylglutaryl-CoA reductase
LOC105233868	39.12	30.559	33.79	–	–	Mevalonate kinase
LOC105230627	20.54	23.40	23.82	+	+	Phosphomevalonate kinase
LOC105224988	14.42	12.11	11.58	–	–	Diphosphomevalonate decarboxylase
LOC105230652	24.54	27.08	26.39	+	+	Farnesyl diphosphate synthase
LOC105225547	70.82	27.06	24.24	−1.41*	−1.50*	Farnesol dehydrogenase
LOC105228451	683.52	151.47	159.65	−2.16*	−2.01*	Farnesoic acid O-methyl transferase
LOC105227166	18.26	3.77	4.05	−2.39*	−2.30*	Juvenile hormone acid O-methyltransferase
Juvenile hormone degradation pathway						
LOC105230544	15.22	2.97	2.07	−2.38*	− 2.87*	Juvenile hormone epoxide hydrolase
LOC105226721	1517.31	230.58	104.88	−2.78*	−3.94*	Juvenile hormone diol kinase
LOC105229968	0.99	0.15	0.034	−2.90*	−5.00*	Juvenile hormone esterase
Juvenile hormone signal transduction pathway						
LOC105228252	22.35	20.74	15.64	–	–	Methoprene-tolerant
LOC105232250	26.87	18.75	14.51	–	–	Steroid receptor coactivator
LOC105224804	23.21	15.81	14.51	–	–	Krüppel homolog 1
Juvenile hormone transporter pathway						
LOC105232271	423.59	2.15	2.09	−7.67*	−7.72*	Juvenile hormone binding protein
LOC105234182	1134.50	7.68	4.28	−7.22*	−8.06*	Juvenile hormone binding protein
LOC109579562	2.75	0.047	0	−6.86*	*	Juvenile hormone binding protein
LOC105232270	2659.47	38.67	19.20	−6.13*	−7.11*	Juvenile hormone binding protein
LOC105232273	2559.46	61.87	23.55	−5.37*	−6.76*	Juvenile hormone binding protein
LOC105234180	19.13	0.63	1.57	−5.13*	−3.70*	Juvenile hormone binding protein
LOC105232274	71.64	2.85	1.11	−4.67*	−6.01*	Juvenile hormone binding protein
LOC105232427	143.97	7.50	3.81	−4.28*	−5.24*	Juvenile hormone binding protein
LOC105226548	11.19	1.13	1.24	−3.40*	−3.22*	Juvenile hormone binding protein
LOC109579964	951.84	145.71	84.49	−2.75*	−3.50*	Juvenile hormone binding protein
LOC105226550	130.82	26.86	33.09	−2.29*	− 2.01*	Juvenile hormone binding protein
LOC105226544	10.54	2.66	2.26	−2.04*	−2.31*	Juvenile hormone binding protein
LOC105231611	10.53	3.77	3.38	−1.51*	−1.71*	Juvenile hormone binding protein

The gene expression level was calculated by RPKM method. “+” represents up-regulation, and “-” represents down-regulation. Asterisks (*) indicate that the gene, whose expression level had more than two folds difference between two stages and False Discovery Rate (FDR) was less than 0.01

### Change in the gene expressions in the chitin degradation and synthesis pathways

**Fig. 6:**
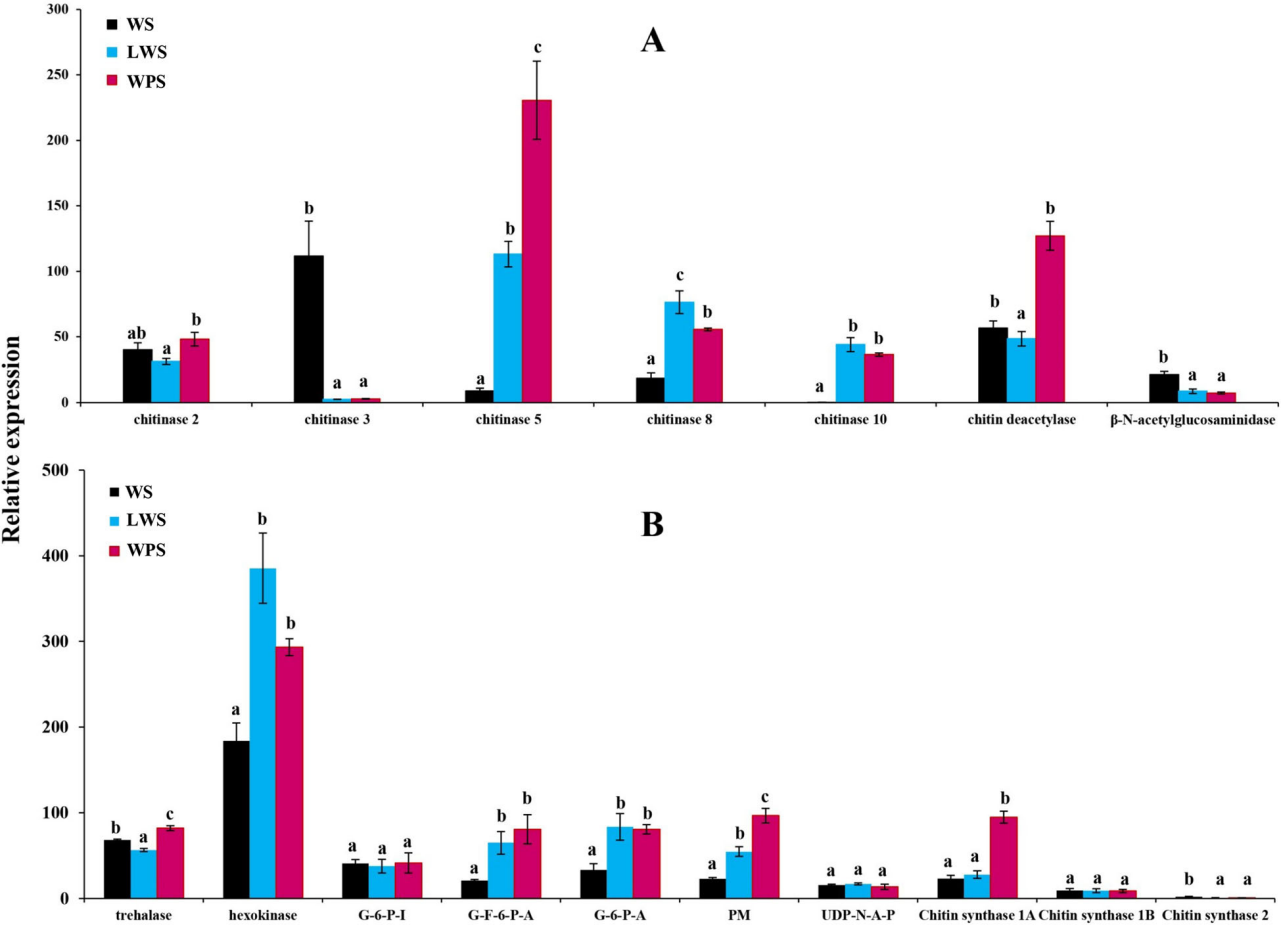
Expression of the selected 17 genes by qRT-PCR. **a** Expression profiles of genes involved in the chitin degradation pathway. **b** Expression profiles of genes involved in the chitin biosynthesis pathway. The data is presented as mean ± SE of three replications. Stages that are statistically different (*P* < 0.05) are marked with a different letter (one-way analysis)

Three serine protease genes were found to be up-regulated and five genes of serine protein inhibitors were down-regulated (Table 3). Interestingly, expression levels of a large number of cuticular protein genes were found to be significantly decreased as well (Table 4). Here, the results of RNA-seq (Table 5) and qRT-PCR (Fig. 6a) showed that the gene expression of four chitinase (chitinase 2, chitinase 5, chitinase 8, and chitinase 10) and chitin deacetylase was significantly increased upon pupariation, indicating their important role in the larval metamorphosis. However, the gene expression patterns of chitinase 3 and β-N-acetylglucosaminidase were different from the above five enzymes, and for these two, mRNA levels were the highest in larval stage, which implied their possible role in the degradation of larval cuticle (Table 5 and Fig. 6a). The expression of genes that are related with the chitin biosynthesis pathway are shown in Table 6 and Fig. 6b, respectively. Based on the qRT-PCR result, we found that most of the genes encoding trehalase, hexokinase, G-F-6-P-A, G-6-P-A, PM, and chitin synthase 1A were significantly up-regulated during the pupariation, except for the genes encoding G-6-P-I, UDP-N-A-P, chitin synthase 1B, and chitin synthase 2 (Fig. 6b).

**Table 3:** Expression level of the serine protease and inhibitor genes during the pupariation in *Bactrocera dorsalis*

Gene ID	WS-FPKM	LWS-FPKM	WPS-FPKM	WS vs LWS	WS vs WPS	Blast nr
Up-regulated						
LOC105227168	0.029	0.48	1.78	4.20	6.08	serine protease nudel
LOC105226969	2.25	36.35	51.87	4.01	4.52	serine protease persephone-like
LOC105224105	76.39	192.55	217.22	1.34	1.51	serine protease easter-like
Down-regulated						
LOC105229202	78.18	5.39	4.37	−4.02	−4.35	serine protease inhibitor
LOC105234027	36.95	4.22	3.32	−3.12	−3.48	serine protease inhibitor
LOC105224656	41.61	5.48	7.52	−2.97	−2.49	serine protease inhibitor
LOC105224655	256.05	68.06	59.24	−1.92	−2.10	serine protease inhibitor
LOC105224653	60.96	17.71	25.93	−1.86	−1.26	serine protease inhibitor

The criteria applied for significance difference are FDR ≤ 0.01, and estimated absolute |log2Ratio| ≥ 1

**Table 4:** Expression level of the cuticular protein genes during the pupariation in *Bactrocera dorsalis*

Gene ID	WS-FPKM	LWS-FPKM	WPS-FPKM	WS vs LWS	WS vs WPS	Blast nr
Down-regulated genes						
LOC105222609	13.95	0	0.16		−6.74	larval cuticle protein A2B
LOC105231490	20.82	0	0.12		−6.82	cuticle protein LPCP-23
LOC105233876	338.64	1.56	1.31	−7.84	−8.05	cuticular proteins
LOC105232796	439.91	5.19	16.49	−6.45	−4.68	endocuticle structural glycoprotein SgAbd-3
LOC105229609	26.99	1.49	1.07	−4.54	−4.85	Larval cuticle protein 9
LOC105228055	2595.35	131.71	44.33	−4.38	−5.93	larval cuticle protein 2-like
LOC105228057	33.16	1.90	1.59	−4.26	− 4.42	larval cuticle protein 2-like
LOC105222608	137.42	10.35	10.72	−3.81	−3.71	larval cuticle protein A2B
LOC105232742	26.60	2.40	0.93	−3.64	−5.17	larval cuticle protein 5-like
LOC105233885	97.17	10.27	7.32	−3.59	− 3.97	Larval cuticle protein 9
LOC105228103	207.33	20.15	31.77	−3.52	−2.54	larval cuticle protein LCP-17
LOC105227146	3.35	0.39	0.28	−3.27	− 3.70	cuticle protein Edg-78E-like
LOC105232751	343.93	20.57	6.29	−3.09	−4.52	endocuticle structural glycoprotein SgAbd-5
LOC105222606	5.84	0.97	0.089	−2.76	−6.94	Larval cuticle protein A2B
LOC105228054	47.12	7.82	12.29	−2.67	− 2.00	larval cuticle protein 2-like
LOC105223825	261.60	59.42	45.43	−2.17	−2.50	cuticle protein 16.8
LOC105228110	802.08	263.54	270.89	−1.68	−1.62	endocuticle structural protein SgAbd-6-like
LOC105232738	23.53	11.22	10.14	−1.23	− 1.36	larval cuticle protein 5-like
LOC105233894	4134.03	1868.15	1170.25	−1.23	−1.91	larval cuticle protein LCP-30
LOC105224878	6.93	3.18	3.14	−1.11	−1.19	Insect cuticle protein
Up-regulated genes						
LOC105225950	0.15	67.39	71.21	9.40	9.57	Endocuticle structural glycoprotein SgAbd-9
LOC105223248	0.98	1.17	1.48	3.72	4.12	Insect cuticle protein

The criteria applied for significance difference are FDR ≤ 0.01, and estimated absolute |log2Ratio| ≥ 1

**Table 5:** Expression level of the chitin degradation related genes during the pupariation in *Bactrocera dorsalis*

Gene ID	WS-FPKM	LWS-FPKM	WPS-FPKM	Blast nr
LOC105224788	36.89	33.07	48.72	chitinase 2
LOC105229469	207.34	6.98	2.60	chitinase 3
LOC105226345	14.70	228.71	586.63	chitinase 5
LOC105232395	13.69	88.89	60.02	chitinase 8
LOC105232357	7.59	58.13	57.74	chitinase 10
LOC105225231	84.25	46.72	182.69	chitin deacetylase
LOC105227151	22.33	5.18	6.92	β-N-acetylglucosaminidase

**Table 6:** Expression levels of the chitin synthesis related genes during pupariation in *Bactrocera dorsalis*

Gene ID	WS-FPKM	LWS-FPKM	WPS-FPKM	Blast nr
LOC105228449	60.60	48.09	71.89	trehalase
LOC105223186	126.74	292.94	189.01	hexokinase type 2
LOC105228090	41.35	43.35	43.93	glucose-6-phosphate isomerase
LOC105231918	26.40	125.20	134.79	glutamine--fructose-6-phosphate aminotransferase
LOC105223703	94.87	138.05	142.47	glucosamine-6-phosphate N-acetyltransferase
LOC105225295	35.89	88.83	103.41	phosphoacetylglucosamine mutase
LOC105233954	60.62	53.57	52.58	UDP-N-acetylhexosamine pyrophosphorylase
LOC105224260	48.31	59.35	196.76	Chitin synthase1A
LOC105226562	3.28	2.86	3.96	Chitin synthase 1B
LOC105224261	2.75	0.99	0.59	Chitin synthase 2

## Discussion

In our previous study, the RNA-seq libraries have been constructed without a reference genome to investigate the gene expression patterns at different developmental stages from eggs to adults (eggs, larvae, pupae, and adults) [3]. Because the lack of an annotated *B. dorsalis* genome, many DEGs were found to encode proteins with unknown function among the different developmental stages, suggesting insufficient knowledge of the molecular mechanisms underlying the development of *B. dorsalis*. Currently, the *B. dorsalis* genome is available online (NCBI Assembly: ASM78921v2) and a total of 11,301 genes were identified during larval pupariation, with a large number of genes changing during the LWS and WPS relative to the WS. Importantly, this work is the first transcriptome exploration of metamorphosis in *B. dorsalis*, which has provided us with this useful gene expression resource to further understand the metamorphosis development in this species.

As the pupa is a relatively stable and immobile stage, the larval stage is the critical stage to obtain energy to complete the metamorphosis [33], and the energy derived from larvae can also make a significant contribution to the reproductive output during their entire life [34, 35]. Therefore, many previous studies have reported that energy metabolic rates declined sharply after pupariation, and are maintained at a low level until the eclosion to the adult [36, 37]. Similarly, the present KEGG analysis also confirmed the previous study in *D. melanogaster* [38], that the oxidative phosphorylation pathway was significantly blocked, indicating less production of energy in LWS and WPS, when comparing to WS in *B. dorsalis*. One of the explanations might be that the WS is a very active stage that aims to find the suitable place for pupariation, on the contrary, the LWS and WPS are both immobile stages, which certainly require less energy supply in these two stages of *B. dorsalis*. Another possible explanation is that energy stores in larvae are limited, and the energy metabolism has to be decreased, otherwise the pupa could simply run out of energy. Moreover, energy stores acquired from the larval stage were retained in the pupa, and would be present to support the newly-eclosed fly adult, as flies do not feed at the beginning of their adult lives [39, 40]. The present result also proves the U-shaped metabolic curve, which is necessary for insect metamorphosis [41].

Interestingly, we also found the expressions of many CYP genes decreased sharply, a similar result has also been reported in *S. litura*, showing that CYP4 and CYP6 groups were predominantly expressed in wandering stage and had a lower expression level in prepupae or pupae stage [18]. The CYP4 and CYP6 family contains the largest number of CYP members, and the genes from these two families are mainly involved in the metabolism of xenobiotics and endogenous toxins in *D. melanogaster* [42] and *Anopheles funestus* [43]. As the puparium offers a physical barrier to protect the immature stages from exposure to xenobiotics, the lower expressions of CYP genes are rational. Some research reported that the CYP3 genes were involved in cuticle formation by the regulation of ecdysone in *D. melanogaster*. Here, the expressions of three CYP3 genes increased significantly, suggesting their possible role in cuticle formation during the pupariation in *B. dorsalis*.

In insects, 20E and JH are the two primary hormones to regulate different biological processes of growth, molting, metamorphosis, and reproduction [16]. The steroid 20E is the molting hormone, which could trigger the molting process and mediate the morphogenetic processes including the metamorphosis in insects [44]. CYP302a1, CYP306a1, CYP307a1 and CYP314a1 are known as the halloween genes, and are involved in the final steps of 20E biosynthetic pathway, catalyzing the conversion of ecdysone to 20E [45]. The RNAi-mediated knockdown of specific halloween genes results in morphological defects such as precocious metamorphosis, impaired nymphs, and cuticle deformities that are accompanied with a decrease of 20E level [45–47]. The 20E signal pathway is composed of nuclear receptors, such as USP, E74, E75, E78, and FTZ-F1 that can strictly control insect metamorphosis [18]. In the current study, we found a higher expression of 20E biosynthesis and signaling pathway genes in WS, as this stage is the proecdysis of *B. dorsalis* larvae, we believe that the higher expression of these genes is crucial to initiate molting process during the pupariation in *B. dorsalis.*

JH is a unique insect hormone, which is synthesized and released from a small group of glands that is located posterior to the brain, called corpora allata (CA) [48]. It has been reported that JH is involved in the regulation of insect molting and metamorphosis by modulating ecdysone action. Previous studies have revealed that changes of JH titers are primarily controlled through the JH synthesis and degradation pathways by the action of specific enzymes [49]. In this study, we found that the JH related genes including synthesis, degradation, signal transduction, and transporter pathways were all down-regulated in the process of metamorphosis, indicating their critical role in the pupariation of *B. dorsalis*. FD is a rate limiting enzyme for JH biosynthesis, which is involved in the oxidation of farnesol to farnesoic acid, suggesting its importance in the regulation of JH titer [50]. Methyl farnesoate is the immediate precursor of JH, and the FAOMT enzyme could catalyze methylation of farnesoic acid into methyl farnesoate, which is the penultimate step of insect JH biosynthesis and thus an important regulator in insect development [51]. As the final step of JH biosynthesis, *JHAOM* is specifically expressed in the CA and its temporal expression strongly correlates with the JH biosynthetic activity, indicating this gene is crucial for the regulation of insect JH biosynthesis [49]. Moreover, knockdown of *JHAOM* in the larval stage of *T. castaneum* by RNAi leads to precocious larval metamorphosis, proving *JHAOM* is required for JH biosynthesis to maintain larval status [52]. Interestingly, the three enzyme genes that are involved in JH degradation pathway were significantly decreased, and the possible reason might be that the down-regulated JH biosynthetic pathway could result in lower JH titter, which was accompanied with the down-regulated JH degradation pathway during the larval metamorphosis in *B. dorsalis*.

*Met*, *Kr-h1* and *SRC* are the key genes in the JH signal transduction pathway [16], and *Met* was regarded as a receptor for JH, and JH-bounded Met could interact with SRC to activate Kr-h1 in *B. mori* [53]. RNAi analysis showed that knockdown of *Met* and *Kr-h1* could both cause precocious metamorphosis, which directly suggested their important role in the larval-pupal transition in *T. castaneum* [54, 55]. JH binding protein (JHBP) could serve as a carrier to bind the JH (JH-JHBP complex) in the hemolymph and release the hormone to target tissues [56]. As expected, the JH signaling and transport pathway were both significantly blocked during the pupariation in *B. dorsalis*. Therefore, according to the results described above, we speculate that 20E and JH regulate metamorphosis via the regulation of specific genes involved in biosynthesis and signaling pathways in *B. dorsalis*.

When *B. dorsalis* developed into the pupariation, lots of cuticular protein genes were down-regulated, whereas some serine protease genes were up-regulated, indicating protein degradation might take place actively in cuticle of *B. dorsalis* during this transformation. Namely, the cuticle proteins were broken down and the chitin was released, so that the chitin could be further degraded [18]. As the vital component of the insect cuticle, the chitin of the cuticle needs to be degraded and replaced by the newly synthesized chitin during the metamorphosis. In the current study, we found that genes encoding chitinase 2, chitinase 5, chitinase 8, chitinase 10, and chitin deacetylase might participate in the chitin degradation as their expression is significantly increased during the pupariation in *B. dorsalis*. Insect chitinases are part of family 18 glycosylhydrolases that could be detected in molting fluid and gut tissues, and regulate the digestion of chitin in the exoskeleton into chitooligosaccharides [57]. Based on the RNAi experiments in *T. castaneum* [57] and *Nilaparvata lugens* [58], silencing of chitinase 5 and chitinase 10 genes resulted in molting defects (insect cannot degrade the old cuticle and then were trapped in their exuviates), which blocked the pupariation. For the chitin deacetylase, the injection of dsRNAs affected the molting of larval-pupal and pupal-adult metamorphosis in *T. castaneum* [59].

The chitin biosynthetic pathway consists of several reactions that are catalyzed by at least eight enzymes, starting with trehalase and ending with the chitin polymer [14]. As expected, the present study showed that most of the chitin biosynthesis pathway genes increased their expression, suggesting their role in the chitin synthesis during the process of pupariation in *B. dorsalis*. Chitin synthase is located at the final step of chitin biosynthetic pathway, and based on sequence similarity and function, the insect chitin synthase could be divided into two types: chitin synthase 1 (consists of two alternative splicing variants, chitin synthase 1A and 1B) and chitin synthase 2 [60]. Importantly, when silencing chitin synthase 1A in *B. dorsalis* by RNAi, the larvae is trapped in its old cuticle and dies without pupation, while when knocking down of chitin synthase 1B, there was no effect on insect development [30]. In the present study, the gene expression pattern of chitin synthase 1A confirmed our previous study that synthase 1A is required for chitin synthesis during the larval metamorphosis in *B. dorsalis* [30].

## Conclusions

In this study, RNA-seq analysis was performed to elucidate the molecular mechanisms of pupariation in *B. dorsalis*. A higher number of genes were found to have a transcriptional profile which was significantly down-regulated during this transition. Especially the genes involved in the oxidative phosphorylation pathway, cytochrome P450s, serine protease, and cuticular proteins, decreased their expressions remarkably. The present study also confirmed that the specific genes in the 20E and JH pathways might be involved in regulating the metamorphosis of *B. dorsalis*. The gene expression of chitinase 2, chitinase 5, chitinase 8, chitinase 10, chitin deacetylase, and chitin synthase 1A suggested their important role in the process of pupariation. Overall, our study provided some basic gene expression data that was helpful to explore the metamorphic mechanisms of *B. dorsalis*, and functional analysis of specific genes is needed to further uncover their possible role in pupariation of *B. dorsalis*.

## Additional files

Additional file 1: **Table S1.** Primers used for quantitative real-time PCR (qRT-PCR). (DOCX 18 kb)

Additional file 2: **Table S2.** Data output quality and mapping rates for the examined sample of *Bactrocera dorsalis*. (DOCX 17 kb)

Additional file 3: **Figure. S1.** Histogram distribution of genes expression level of each sample. X-axis is FPKM value (the coordinate has been changed by logarithm for better view). Y-axis is the probability density of corresponding FPKM. (JPG 469 kb)

Additional file 4: **Table S3.** All genes FPKM value during the pupariation in *Bactrocera dorsalis*. (XLSX 1346 kb)

Additional file 5: **Figure. S2.** Numbers of differentially expressed genes between late wandering stage and white puparium stage (DEGs, FDR < 0.01 and |log2 ratio| ≥ 1), the up-regulated genes were represented in red dot and down-regulated genes in green dot. (JPG 236 kb)

Additional file 6: **Figure. S3.** The annotated of differentially expressed genes based on different databases. Nr (NCBI non-redundant protein sequences); Pfam (Protein family); COG (Clusters of Orthologous Groups of proteins); Swiss-Prot (A manually annotated and reviewed protein sequence database); KEGG (Kyoto Encyclopedia of Genes and Genomes); eggNOG (evolutionary genealogy of genes: Non-supervised Orthologous Groups); GO (Gene Ontology). (JPG 762 kb)

Additional file 7: **Table S4.** Detail information about DEGs for wandering stage and late wandering stage in *Bactrocera dorsalis*. (XLSX 634 kb)

Additional file 8: **Table S5.** Detail information about DEGs for wandering stage and white puparium stage in *Bactrocera dorsalis*. (XLSX 683 kb)

Additional file 9: **Figure. S4.** GO classification of the differentially expressed genes (DEGs) in wandering stage vs. late wandering stage. (JPG 1149 kb)

Additional file 10: **Figure. S5.** GO classification of the differentially expressed genes (DEGs) in late wandering stage vs. white puparium stage. (JPG 1104 kb)

Additional file 11: **Figure. S6.** COG classification of the differentially expressed genes (DEGs). (A) COG classification of DEGs in wandering stage (WS) vs. late wandering stage. (B) COG classification of DEGs in WS vs. white puparium stage. (JPG 912 kb)

Additional file 12: **Table S6.** The detail FPKM value of 20-hydroxyecdysone biosynthesis and signaling related genes during the pupariation in *Bactrocera dorsalis*. (XLSX 12 kb)

## Abbreviations

20E: 20-hydroxyecdysone
ATP: Adenosine triphosphate
BR-C: Broad complex
CA: Corpora allata
COG: Clusters of orthologous groups of proteins
CYP: Cytochrome P450
DEGs: Differentially expressed genes
DEGs: differentially expressed genes
FAOMT: Farnesoic acid O-methyl transferase
FD: Farnesol dehydrogenase
FDR: False discovery rate
FPKM: Fragment per kilobase of exon model per million mapped reads
FTZ-F1: Fushi tarazu factor 1
G-6-P-A: Glucosamine-6-phosphate N-acetyltransferase
G-6-P-I: Glucose-6-phosphate isomerase
G-F-6-P-A: Glutamine--fructose-6-phosphate aminotransferase
GO: Gene ontology
JH: Juvenile hormone
JH: Juvenile hormone
JHAOM: Juvenile hormone acid O-methyltransferase
JHBP: Juvenile hormone binding protein
KEGG: Kyoto encyclopedia of genes and genomes
Kr-h1: Krüppel homolog 1
LWS: Late wandering stage
Met: Methoprene-tolerant
Nr: NCBI non-redundant protein sequences
Nt: NCBI non-redundant nucleotide sequences
Pfam: Protein family
PM: Phosphoacetylglucosamine mutase
SRC: Steroid receptor coactivator
Swiss-Prot: A manually annotated and reviewed protein sequence database
UDP-N-A-P: UDP-N-acetylglucosamine pyrophosphorylase
USP: Ultraspiracle protein
WPS: White puparium stage
WS: Wandering stage
