# Supplementary material for: RNA-seq analysis of gene expression changes during pupariation in Bactrocera dorsalis (Hendel) (Diptera: Tephritidae)
Source: BMC Genomics. 2018 Sep 21;19:693. doi: 10.1186/s12864-018-5077-z (PMC6150976; doi:10.1186/s12864-018-5077-z)
Supplement: Supplementary file 1 — Table S1. Primers used for quantitative real-time PCR (qRT-PCR). (DOCX 18 kb) [file 12864_2018_5077_MOESM1_ESM.docx]

**Table S1** Primers used for quantitative real-time PCR (qPCR).

| Target | Direction | Sequence 5’ to 3’ |
| --- | --- | --- |
| *chitinase 2* | Forward | ATCTGCTGAAAATGGGTGCG |
|  | Reverse | TCCTCACGAGTAAAGGGACC |
| *chitinase 3* | Forward | GCCGCTAATTCAACGTCCAT |
|  | Reverse | CGACATTGCTGAGAGATCGC |
| *chitinase 5* | Forward | CCAACATCATCAAGCAGCGA |
|  | Reverse | TTCTGACAATCTGCATGCGG |
| *chitinase 8* | Forward | TTGCAGGACCCAACGATACT |
|  | Reverse | AGTGTGAAAGAGCGTCCGTA |
| *chitinase 10* | Forward | ACGCCATGCAACTGTTGATT |
|  | Reverse | CGCCTACTAAAGTGCTGCTG |
| *chitin deacetylase* | Forward | TGCGTCACAATTTCAACCGT |
|  | Reverse | ACGTGTCAGCATCTCTTCGA |
| *β-N-acetylglucosaminidase* | Forward | GGACAATTCGATCCCACTGC |
|  | Reverse | ACGGATACCTTCACTCGCAT |
| *trehalase* | Forward | CAATTGTGCCCGTCGATCTT |
|  | Reverse | CCGGCTTCTTCATTCCAGTG |
| *hexokinase* | Forward | CGCATGCAAGTGTGACGATA |
|  | Reverse | ATGCCGATTTGTACGCGAAA |
| *glucose-6-phosphate isomerase* | Forward | GTGGCCCGAACGTGTAATTT |
|  | Reverse | TTCAGATTGACGCCTCCCTT |
| *glutamine--fructose-6-phosphate aminotransferase* | Forward | CATTCCGTGAGCTTGTCGAG |
|  | Reverse | CTGATCAATGGACAGGCGTG |
| *glucosamine-6-phosphate N-acetyltransferase* | Forward | AAGATCTCTGGGTGCGCAAT |
|  | Reverse | CAATACCTTCGGCGACAGTG |
| *phosphoacetylglucosamine mutase* | Forward | AAGGGCTGGGACGTTAAAGA |
|  | Reverse | TGTCACACAAACACGCTCTG |
| *UDP-N-acetylhexosamine pyrophosphorylase* | Forward | GGCTATCGTCTCAATCCGGA |
|  | Reverse | AATCGTTGCATACACGGTGG |
| *Chitin synthase 1A* | Forward | TGAAATATGCACGCCGTCTG |
|  | Reverse | TCGCGACCCTCTAAATGACA |
| *Chitin synthase 1B* | Forward | GAACTCGGTAATGGCGGAAC |
|  | Reverse | GTTGGCGTTGAACACATCCT |
| *Chitin synthase 2* | Forward | AGACGCGGCACAAGAAATAC |
|  | Reverse | GTTGCTGTCGTCGCCATTTA |
| *α*-*Tubulin* (qRCR) | Forward | CGCATTCATGGTTGATAACG |
|  | Reverse | GGGCACCAAGTTAGTCTGGA |
