# Supplementary material for: RNA-seq analysis of gene expression changes during pupariation in Bactrocera dorsalis (Hendel) (Diptera: Tephritidae)
Source: BMC Genomics. 2018 Sep 21;19:693. doi: 10.1186/s12864-018-5077-z (PMC6150976; doi:10.1186/s12864-018-5077-z)
Supplement: Supplementary file 2 — Table S2. Data output quality and mapping rates for the examined sample of Bactrocera dorsalis. (DOCX 17 kb) [file 12864_2018_5077_MOESM2_ESM.docx]

**Table S2** Data output quality and mapping rates for the examined samples of *Bactrocera dorsalis*.

| **Samples** | **Clean reads** | **Clean**  **Data Rate (%)** | **Q30**  **(%)** | **GC**  **(%)** | **Genome**  **Mapping Ratio**  **(%)** |
| --- | --- | --- | --- | --- | --- |
| WS-1 | 31,067,211 | 99.60 | 92.19 | 41.97 | 74.44 |
| WS-2 | 24,274,808 | 99.35 | 91.74 | 41.96 | 70.88 |
| WS-3 | 25,972,280 | 99.43 | 91.63 | 41.41 | 72.16 |
| LWS-1 | 30,596,685 | 99.44 | 91.38 | 41.09 | 73.77 |
| LWS-2 | 31,470,679 | 99.42 | 91.68 | 41.34 | 70.09 |
| LWS-3 | 25,649,371 | 99.37 | 91.33 | 41.10 | 71.59 |
| WPS-1 | 26,334,526 | 99.36 | 91.30 | 41.79 | 73.56 |
| WPS-2 | 28,412,458 | 99.43 | 91.54 | 41.67 | 73.38 |
| WPS-3 | 25,597,510 | 99.44 | 91.73 | 41.51 | 70.15 |

Clean Data Rate (%) = Clean Reads Number / Raw Reads Number.
