# Supplementary figures and images for: RNA-seq analysis of gene expression changes during pupariation in Bactrocera dorsalis (Hendel) (Diptera: Tephritidae)
Source: BMC Genomics. 2018 Sep 21;19:693. doi: 10.1186/s12864-018-5077-z (PMC6150976; doi:10.1186/s12864-018-5077-z)

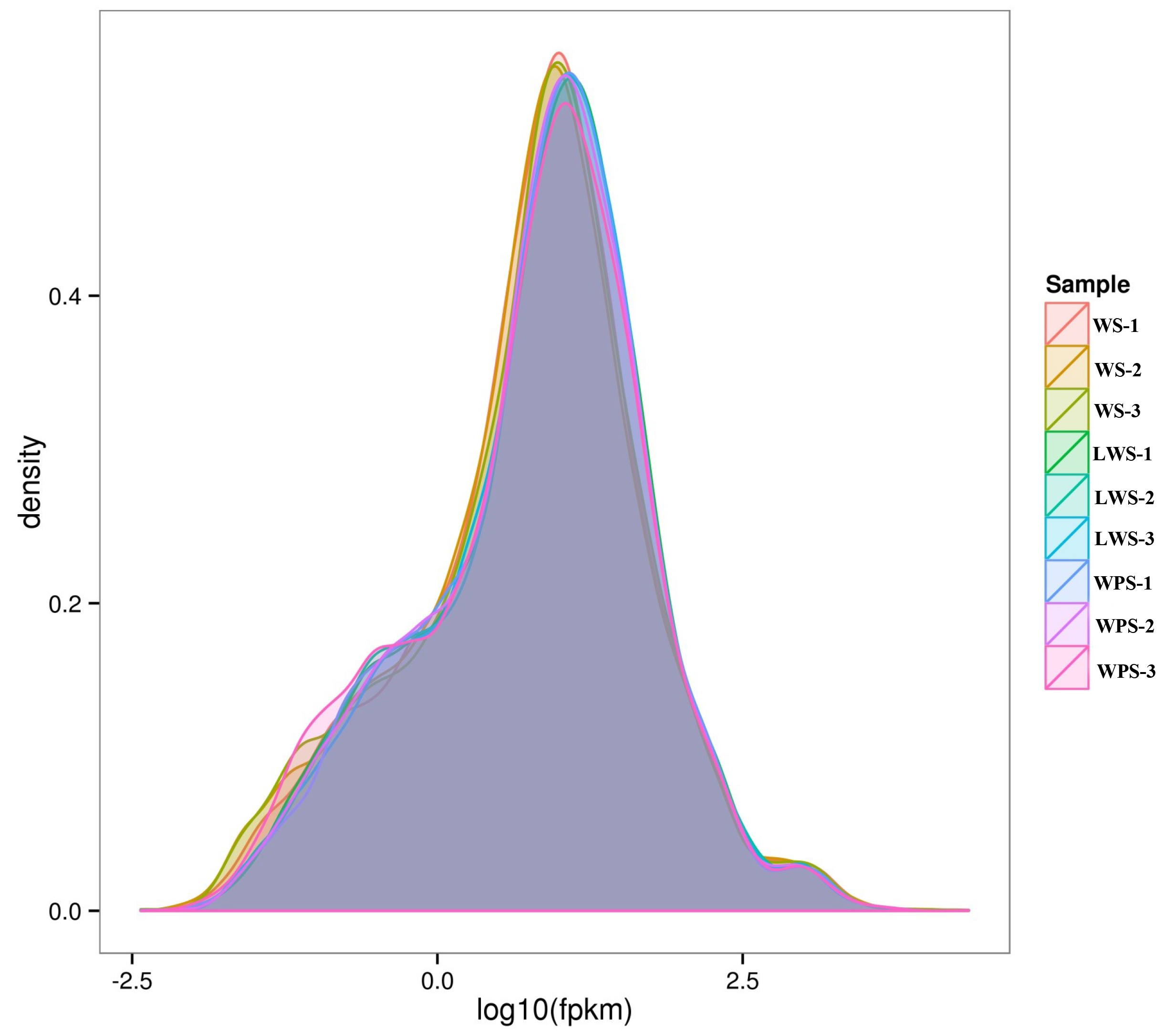

Supplement: Supplementary file 3 — Figure. S1. Histogram distribution of genes expression level of each sample. X-axis is FPKM value (the coordinate has been changed by logarithm for better view). Y-axis is the probability density of corresponding FPKM. (JPG 469 kb) [file 12864_2018_5077_MOESM3_ESM.jpg]

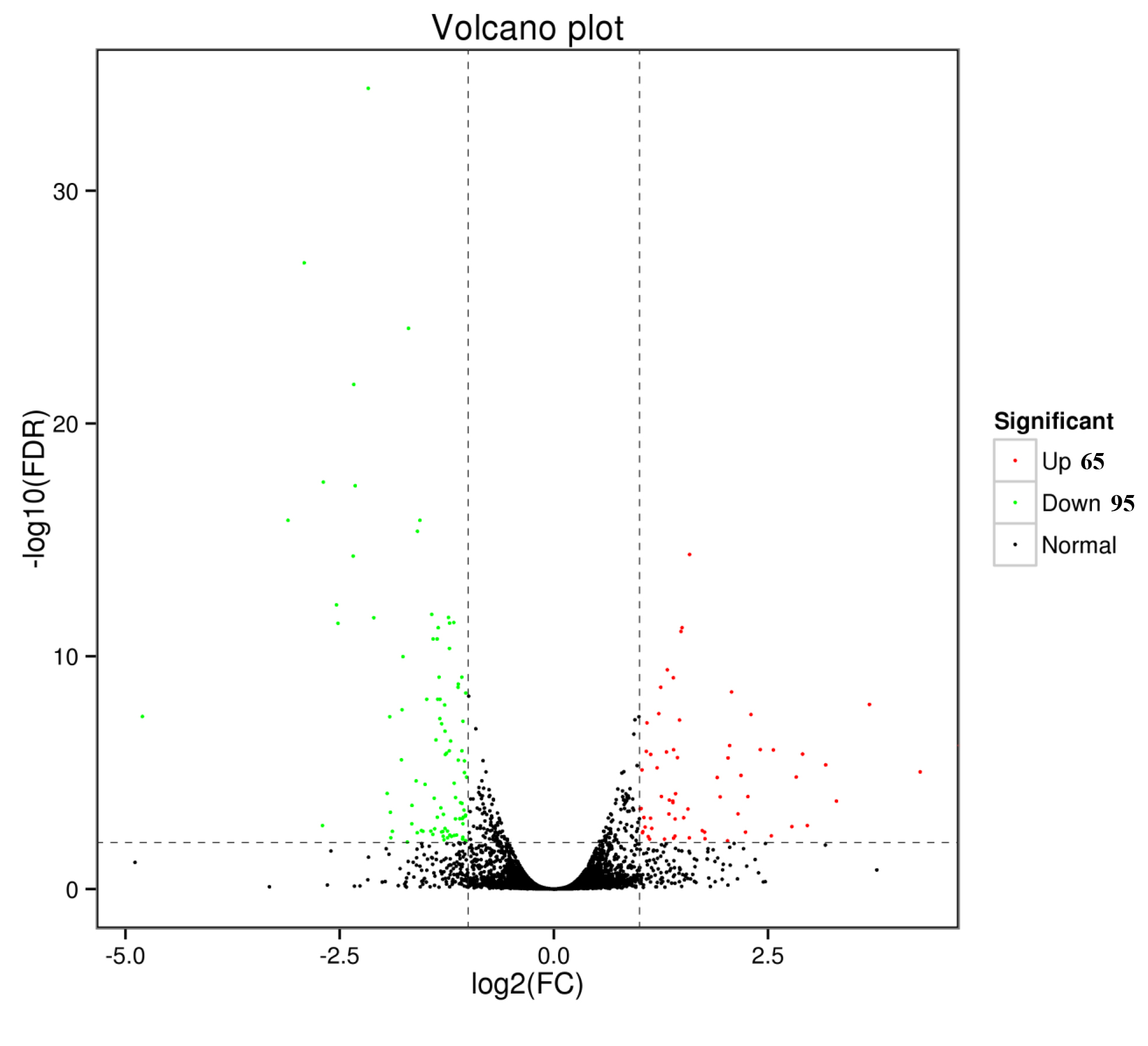

Supplement: Supplementary file 5 — Figure. S2. Numbers of differentially expressed genes between late wandering stage and white puparium stage (DEGs, FDR < 0.01 and |log2 ratio| ≥ 1), the up-regulated genes were represented in red dot and down-regulated genes in green dot. (JPG 236 kb) [file 12864_2018_5077_MOESM5_ESM.jpg]

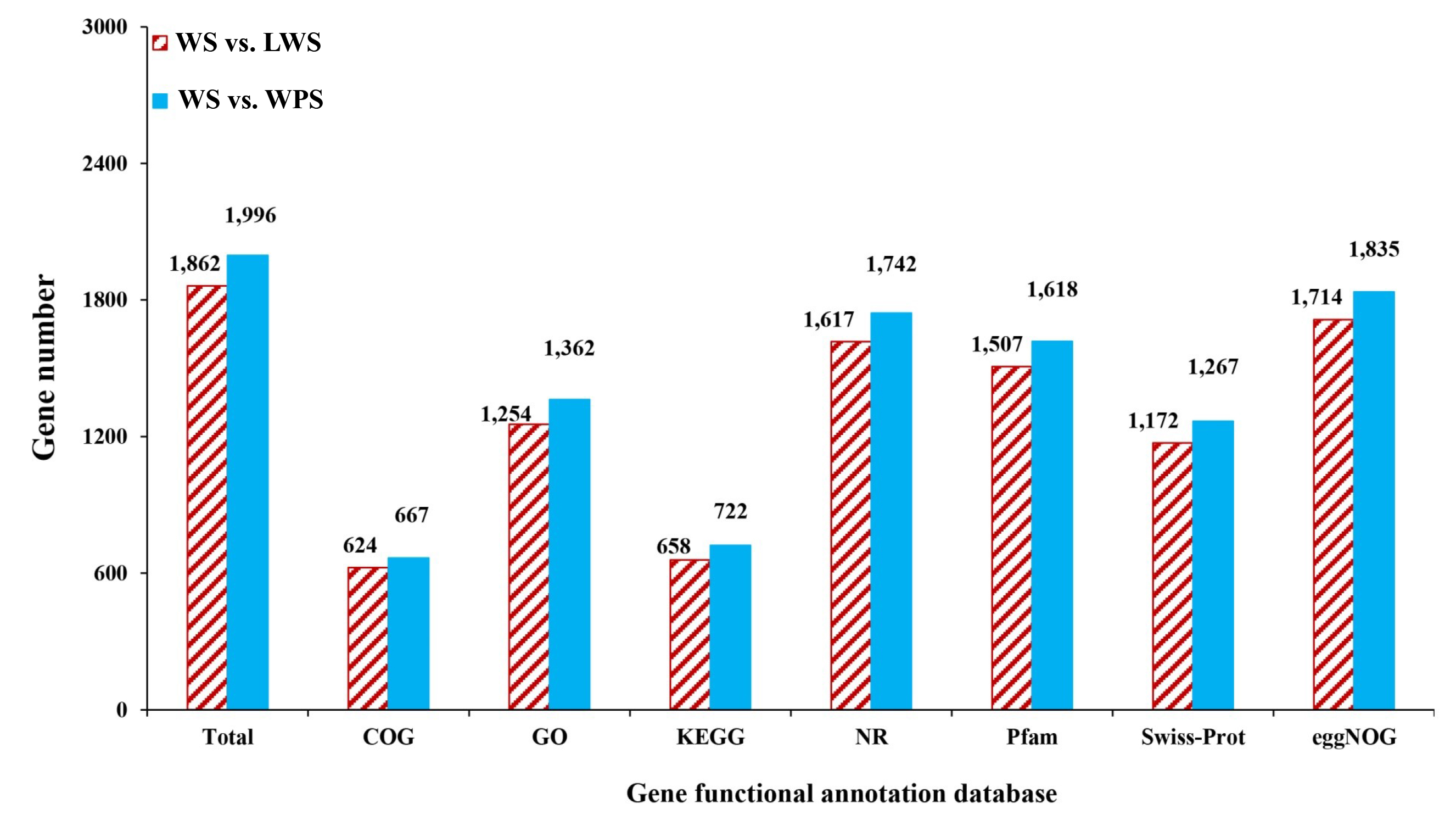

Supplement: Supplementary file 6 — Figure. S3. The annotated of differentially expressed genes based on different databases. Nr (NCBI non-redundant protein sequences); Pfam (Protein family); COG (Clusters of Orthologous Groups of proteins); Swiss-Prot (A manually annotated and reviewed protein sequence database); KEGG (Kyoto Encyclopedia of Genes and Genomes); eggNOG (evolutionary genealogy of genes: Non-supervised Orthologous Groups); GO (Gene Ontology). (JPG 762 kb) [file 12864_2018_5077_MOESM6_ESM.jpg]

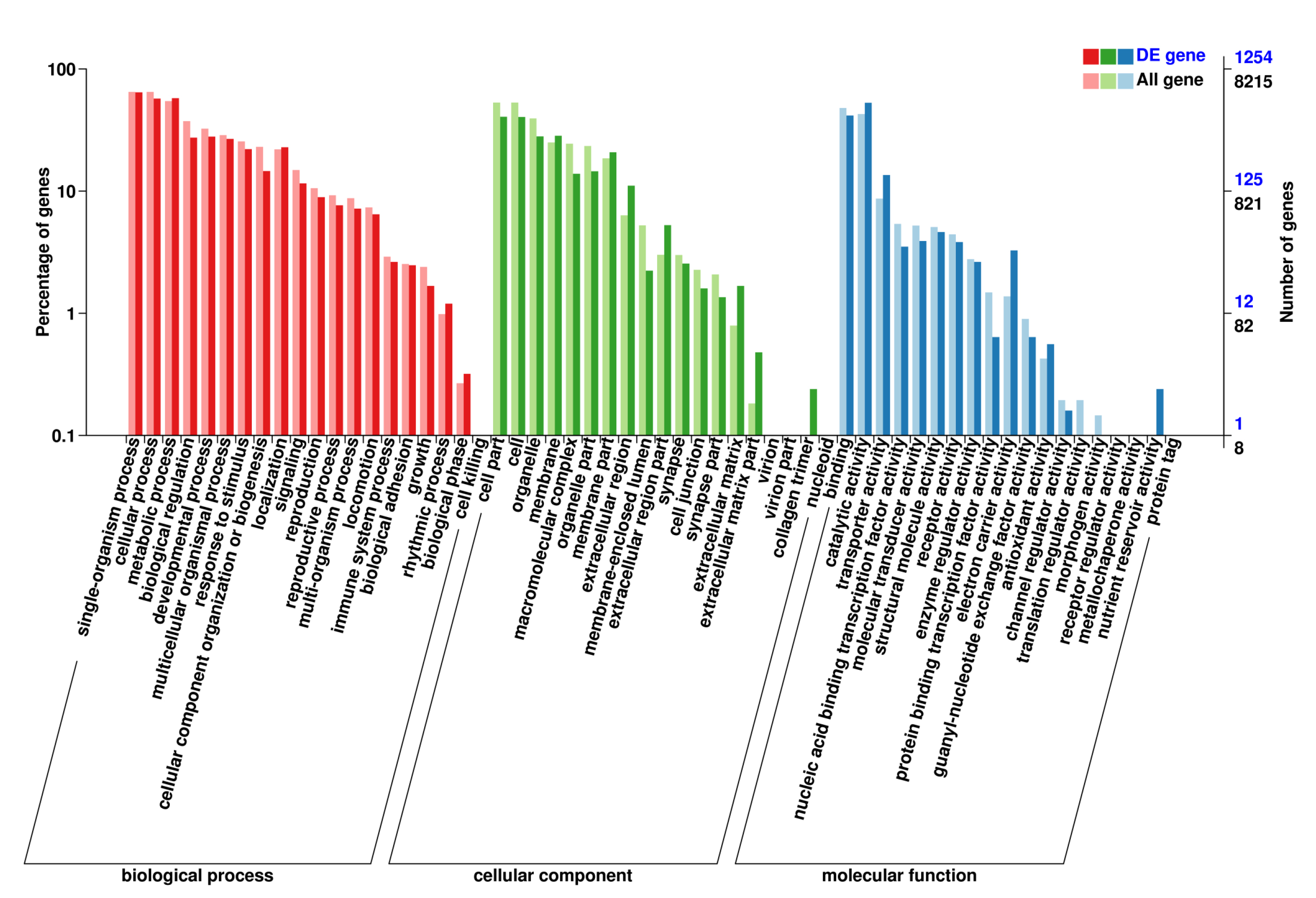

Supplement: Supplementary file 9 — Figure. S4. GO classification of the differentially expressed genes (DEGs) in wandering stage vs. late wandering stage. (JPG 1149 kb) [file 12864_2018_5077_MOESM9_ESM.jpg]

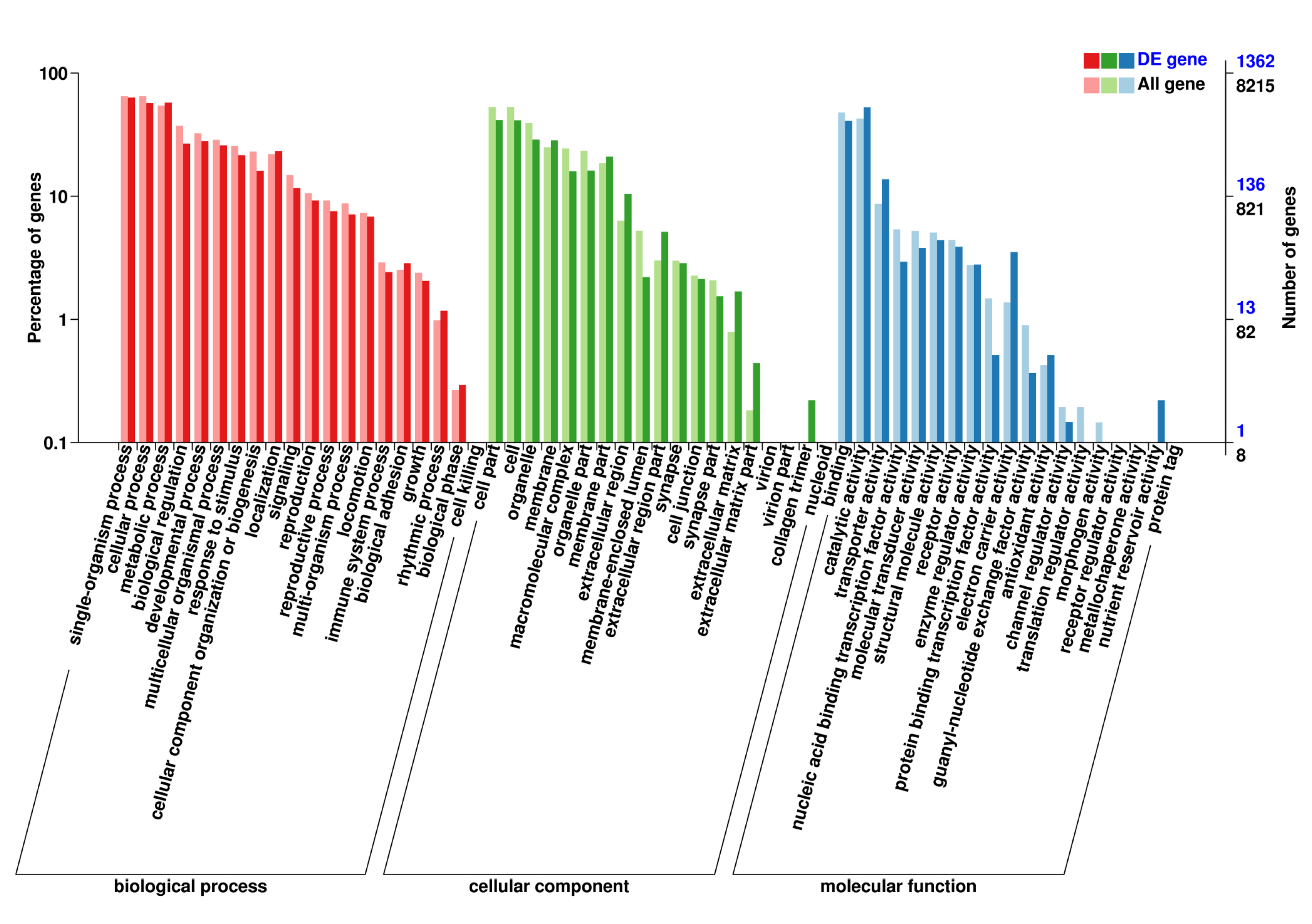

Supplement: Supplementary file 10 — Figure. S5. GO classification of the differentially expressed genes (DEGs) in late wandering stage vs. white puparium stage. (JPG 1104 kb) [file 12864_2018_5077_MOESM10_ESM.jpg]

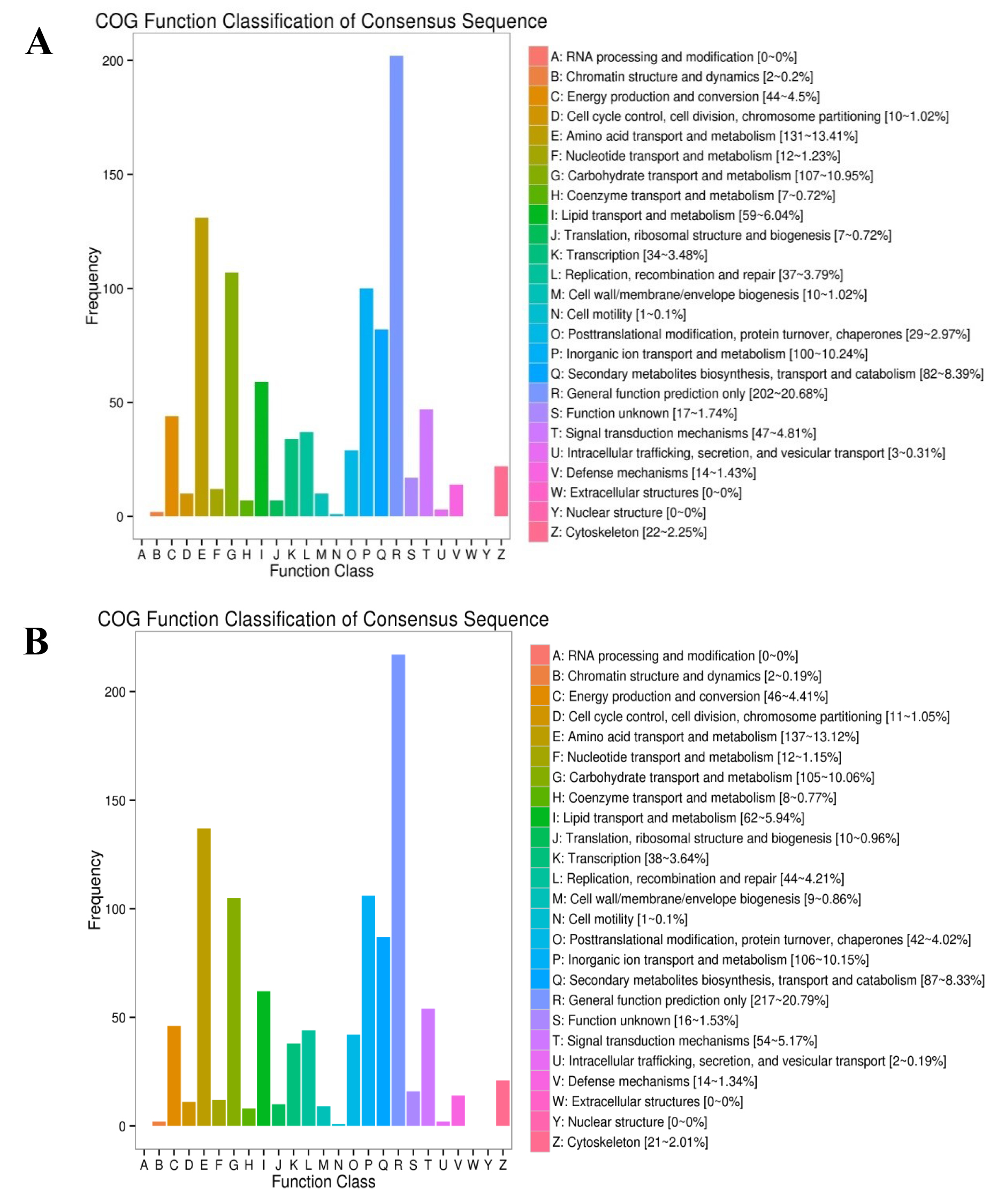

Supplement: Supplementary file 11 — Figure. S6. COG classification of the differentially expressed genes (DEGs). (A) COG classification of DEGs in wandering stage (WS) vs. late wandering stage. (B) COG classification of DEGs in WS vs. white puparium stage. (JPG 912 kb) [file 12864_2018_5077_MOESM11_ESM.jpg]
